# The Hospital de Câncer de Barretos Registry: an analysis of cancer survival at a single institution in Brazil over a 10-year period

**DOI:** 10.1186/1756-0500-6-141

**Published:** 2013-04-10

**Authors:** Estela Cristina Carneseca, Edmundo Carvalho Mauad, Marcos Aurélio Alves de Araujo, Rafael Macrina Dalbó, Adhemar Longatto Filho, Vinicius de Lima Vazquez

**Affiliations:** 1 Hospital Cancer Registry - Institute for Research and Education, Hospital de Câncer de Barretos, São Paulo, Barretos, Brazil; 2 Director of Hospital de Câncer de Barretos, Barretos, São Paulo, Brazil; 3 Laboratory of Medical Investigation (LIM) 14, Faculty of Medicine, University of São Paulo, São Paulo, Brazil; 4 Life and Health Sciences Research Institute (ICVS), School of Health Sciences, University of Minho, Braga, Portugal; 5 ICVS/3B’s - PT Government Associate Laboratory, Braga/Guimarães, Portugal; 6 Department of Surgery, Hospital de Câncer de Barretos, Barretos, São Paulo, Brazil; 7 Molecular Oncology Research Center - Institute for Research and Education, Hospital de Câncer de Barretos, Barretos, São Paulo, Brazil; 8 Hospital de Câncer de Barretos, 1331 Antenor Duarte Villella Street, Zip Code: 14784-400 Barretos, São Paulo, Brazil

**Keywords:** Cancer hospital registry, Descriptive study, Survival, Cancer, Follow-up, Data quality

## Abstract

**Background:**

Epidemiological studies that describe cancer survival statistics at specific hospitals are scarce. Cancer registries, which are collections of cancer patient characteristics, treatment and outcome data, help determine quality of care and treatment indicators.

**Methods:**

This study analysed data from patients treated between 2000 and 2009 at the Hospital de Câncer de Barretos, a referral cancer hospital in Brazil. The analysis included all cases among the nine most common types of cancer diagnosed between 2000 and 2009. The main characteristics of the patients, tumours, treatment procedures and survival were described and discussed. The five-year survival rate of patients with cancer diagnosed between 2000 and 2005 were estimated using Kaplan-Meier methods. Multivariable analysis was performed using Cox proportional hazards regression.

**Results:**

It was analyzed 42,825 cancer cases relating to the nine primary locations in more frequent at the institution. Most of the patients were men (52.8%) and over the age of 60 years (65.1%). Approximately 1% of the treated cancers were not staged, and 0.4% lacked follow-up data. Excluding nonmelanoma skin cancer, the most common tumours were prostate and breast cancer, which were mainly diagnosed at early stages. Five-year survival for these cancers were 78.2% and 74.8%, respectively.

**Conclusions:**

During this ten-year period, the Hospital de Câncer de Barretos Registry collected, processed and analysed data related to all cases treated at the institution, providing relevant information about patient characteristics and survival.

## Background

Cancer is the principal cause of death in developed countries and the second leading cause of death in developing countries. There were an estimated 12.7 million new cancer cases and 7.6 million deaths globally in 2008, according to the International Agency for Research on Cancer (IARC) [1]. Breast cancer has the highest incidence in women, while lung cancer in men has the highest mortality. The Brazilian Health Authorities at the National Cancer Institute (INCA) estimate that approximately 490,000 new cancer cases in Brazil were diagnosed in 2011 [2]. The most common cancers are those that arise in lung, breast, colon and rectum, stomach, prostate, liver, cervical and oesophageal tissues. The high incidences, mortality rates, and prevalence of these common malignancies are associated with socioeconomic factors, individual behaviour, and infectious and microenvironment risk factors. Approximately 25 million persons worldwide were living with cancer in 2002 [3].

The idea of creating registries for certain diseases arose from the need to comprehensively characterize the diseases in order to help identify their causes and, consequently, the most effective interventions [4]. Information regarding disease incidence, prevalence and mortality are critical for determining prevention strategies and for supporting public health initiatives that aim to decrease the incidence of the disease. Such initiatives illustrate the usefulness of information from population-based cancer registries [4]. Most descriptive cancer studies make use of these sources, rather than using data from hospital cancer registries. The cancer registries at general hospitals or cancer hospitals collect data related to the diagnosis, treatment and outcome of patients with malignant tumours. A hospital registry is essential for assessing the quality of information, which reflects the quality of the medical care performed in these hospitals. Careful statistical analysis and interpretation of the data allow the hospital to monitor treatment effectiveness and help the professional staff make treatment decisions and follow-up their cases [4]. Currently, in South America, most of the hospital data related to patient mortality are underestimated; survival rates not known. For cancer patients, this is mainly due to a lack of information, or poor quality information, regarding patient medical history, cancer type, cancer staging and place of death. This lack of accurate and thorough records currently prevents the comparison of data in different hospital registries [5]. Accordingly, the main objectives of this study were to characterize the major cancers treated at the Hospital de Câncer de Barretos, from 2000 to 2009.

The city of Barretos, where is located the Fundação Pio XII – Hospital de Câncer de Barretos, is situated on northern region of the São Paulo State, Brazil. With approximately 112000 inhabitants [6], its economy is essentially composed of agriculture (primarily, by cane sugar, oranges and soybeans), trade and services. In addition to dairy industries, rubber, citrus juices and artefacts, slaughterhouses in the region are important ways to exporting beef to the domestic and foreign market. This characteristic of the economy is that it gives a characteristic rural to Barretos, unlike other municipalities upcountry in the state.

The Hospital de Câncer de Barretos receives cancer patients from all regions of Brazil, with approximately 1000 medical visits per day. The hospital is currently recognized as one of the main references for the treatment of cancer in the country, it has also focused in the last years over the field of research and medical education. The data obtained by the institutional Cancer Hospital Registry allowed this study, so that a summary of the information about the tumor (site and stage at first examination) and patient survival could be made.

## Methods

This retrospective study analysed data retrieved from the Hospital de Câncer de Barretos Registry. In this Registry, patient medical records are entered into a database according to a standardized procedure. Personal information, such as sex, age group and education level, as well as tumour characteristics such as site, date of diagnosis, disease stage and initial treatment, were extracted from the database and analysed.

The study included all cases among the nine most common types of malignant tumor diagnosed at the Hospital de Câncer de Barretos between 2000 and 2009. In addition, we analyzed only the cases effectively treated in the institution and disease staging classified by TNM. A consent term was assigned by each patient participating in the study in your admission at the institution and the study has been approved by the Research Ethics Committee of Hospital de Câncer de Barretos.

Initially we conducted a preliminary analysis of all available variables; subsequently, we determined the five-year survival for cases diagnosed between 2000 and 2005 using Kaplan-Meyer methods. The survival rates were estimated in months, and survival was defined as the period from the date of the first hospital consultation to the date of death or the date at which information was last obtained from the patient. For the analysis, the event of interest was death related to cancer. Cases that were alive or dead from other causes were censored. It is understood by death from other causes, death that occurred due to diseases other than cancer such as heart disease or other medical conditions. Such information was obtained through direct consultation to the death certificate or medical records. Multivariable Cox proportional hazards regression models were used to estimate hazard ratios (HRs) and 95% confidence intervals (CIs) with adjustment for sex, age group and disease stage. Possible confounders such as initial treatment were also included as variable in this analysis [7]. All analyses were stratified for primary site of cancer. The data were exported to SPSS for Windows® v. 17.0 (Inc., Chicago, IL, USA) for statistical analysis. The level of statistical significance was set at 0.05 for all analyzes.

We used the International Classification of Diseases for Oncology (ICD-O) [8] for classification and coding of the topography and histology of the tumours. Tumours were staged using the TNM Classification of Malignant Tumours as proposed by the International Union against Cancer (UICC) [9].

## Results

During the study period from 2000–2009, 67,010 new analytical cases were registered at the Hospital de Câncer de Barretos in Brazil. The nine most common types of malignant tumor were nonmelanoma skin cancers, prostate, breast, cervical, colorectal, lung, stomach, oesophagus and oral cavity. They were diagnosed in 73.5% of the cases registered in this period (n = 49,269). Of these, we analyzed only 42,825 cases that were effectively staged using TNM classification and treated at the institution. These cases included 20,642 cases of nonmelanoma skin cancer (48.2%), 6508 cases of prostate cancer (15.2%), 5257 cases of breast cancer (12.3%), 2631 cases of cervical cancer (6.1%), 2549 cases of colorectal cancer (6.0%), 2067 cases of lung cancer (4.8%), 1467 cases of stomach cancer (3.4%), 853 cases of oesophagus cancer (2.0%) and 851 cases of oral cavity tumours (2.0%).

There were more men in the study population (52.8%, n = 22,604), and more patients were older than 60 years (65.1%, n = 27,885). Regarding education level, the patients were predominantly illiterate or had an incomplete primary school education. About 30% of patients lived outside of São Paulo State. However, as patients residing near Barretos (cities in the São Paulo State), most patients living far from the institution was diagnosed with advanced cancer (49.7% versus 48.6%, ignoring cases of nonmelanoma skin). The distribution of cases according to primary malignant neoplasm, sex, age group and clinical stage are shown in Table 1.

**Table 1:** Patient and tumour characteristics

**Primary site**	**Sex**		**Age group**		**Disease stage**				
	**Male**	**Female**	**<60**	**60+**	**0**	**I**	** II**	**III**	**IV**
	**n (%)**	**n (%)**	**n (%)**	**n (%)**	**n (%)**	**n (%)**	**n (%)**	**n (%)**	**n (%)**
Nonmelanoma skin	10858	9784	5163	15479	813	17928	1617	261	23
	(52.6)	(47.4)	(25.0)	(75.0)	(3.9)	(86.9)	(7.8)	(1.3)	(0.1)
Prostate	6508	-	1011	5497	0	457	3291	1685	1075
	(100.0)		(15.5)	(84.5)	(0.0)	(7.0)	(50.6)	(25.9)	(16.5)
Breast	21	5236	3385	1872	450	816	2048	1470	473
	(0.4)	(99.6)	(64.4)	(35.6)	(8.6)	(15.5)	(38.9)	(28.0)	(9.0)
Cervical	-	2631	1924	707	1091	413	422	558	147
		(100.0)	(73.1)	(26.9)	(41.5)	(15.7)	(16.0)	(21.2)	(5.6)
Colorectal	1341	1208	1187	1362	19	417	862	652	599
	(52.6)	(47.4)	(46.6)	(53.4)	(0.7)	(16.4)	(33.8)	(25.6)	(23.5)
Lung	1431	636	757	1310	0	97	52	950	968
	(69.2)	(30.8)	(36.6)	(63.4)	(0.0)	(4.7)	(2.5)	(46.0)	(46.8)
Stomach	1031	436	610	857	9	190	181	345	742
	(70.3)	(29.7)	(41.6)	(58.4)	(0.6)	(13.0)	(12.3)	(23.5)	(50.6)
Oesophagus	737	116	447	406	6	20	195	348	284
	(86.4)	(13.6)	(52.4)	(47.6)	(0.7)	(2.3)	(22.9)	(40.8)	(33.3)
Oral cavity	677	174	456	395	13	113	160	189	376
	(79.6)	(20.4)	(53.6)	(46.4)	(1.5)	(13.3)	(18.8)	(22.2)	(44.2)

**Figure 1:**
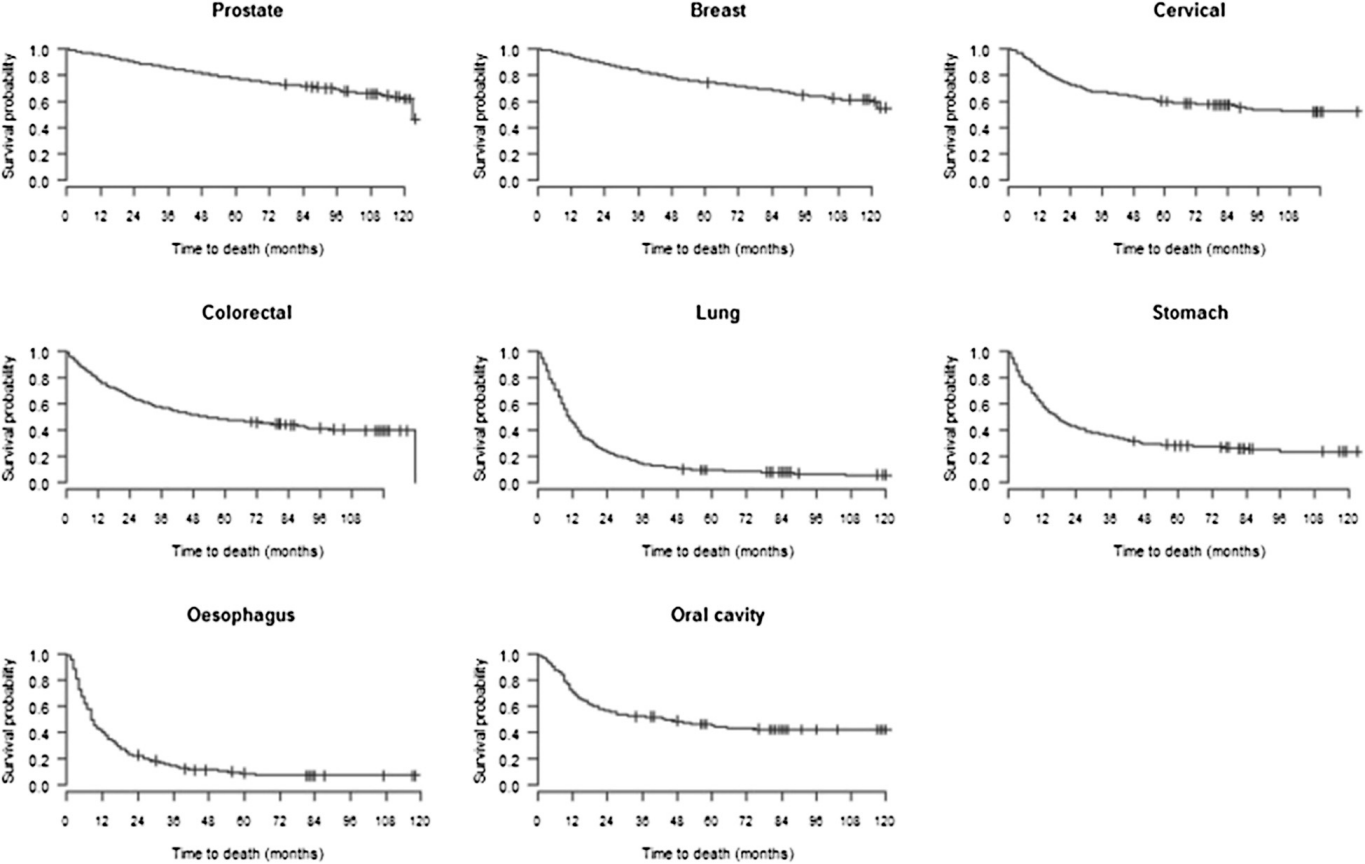
Overall survival curves for different types of cancer as estimated using Kaplan-Meier methods.

The majority of cases of prostate, breast or cervical cancers were at less advanced stages. In contrast, patients with lung, stomach, oesophagus and oral cavity tumours generally were more advanced in stage at the time of diagnosis. This is reflected in the survival curves shown in Figure 1.

Regarding the initial treatment, surgery alone was the most commonly indicated therapy, with the specific treatment depending on the type of malignant neoplasm and its clinical stage. Table 2 shows the initial treatments according to tumour site and clinical stage. The majority of stages 0–II tumours were primarily treated surgically.

**Table 2:** Tumour site, clinical stage and initial treatment

**Primary site**	**Disease stage**	**Initial treatment**					**Total**
		**Surgery**	**Chemotherapy with or without surgery**	**Radiotherapy with or without surgery**	**Chemotherapy + Radiotherapy**	**Other combinations of treatment**	
		**n (%)**	**n (%)**	**n (%)**	**n (%)**	**n (%)**	
Nonmelanoma skin	0	673 (82.8)	0 (0.0)	140 (17.2)	0 (0.0)	0 (0.0)	813
	I	15349 (85.6)	1 (0.01)	2569 (14.33)	1 (0.01)	8 (0.04)	17928
	II	1309 (81.0)	0 (0.0)	307 (19.0)	0 (0.0)	1 (0.1)	1617
	III	158 (60.5)	1 (0.4)	98 (37.6)	3 (1.1)	1 (0.4)	261
	IV	10 (43.4)	1 (4.4)	11 (47.8)	1 (4.4)	0 (0.0)	23
Prostate	I	191 (41.8)	0 (0.0)	206 (45.1)	1 (0.2)	59 (12.9)	457
	II	1365 (41.5)	203 (6.2)	1446 (44.0)	8 (0,2)	269 (8.1)	3291
	III	223 (13.2)	176 (10.5)	681 (40.4)	15 (0.9)	590 (35.0)	1685
	IV	0 (0.0)	61 (5.7)	160 (14.9)	6 (0.5)	848 (78.9)	1075
Breast	0	256 (57.0)	0 (0.0)	132 (29.4)	7 (1.6)	54 (12.0)	449
	I	219 (26.8)	193 (23.6)	69 (8.4)	37 (4.5)	299 (36.7)	817
	II	446 (21.8)	737 (36.0)	92 (4.5)	52 (2.5)	721 (35.2)	2048
	III	119 (8.1)	919 (62.5)	23 (1.5)	26 (1.8)	383 (26.1)	1470
	IV	14 (3.0)	232 (49.0)	35 (7.4)	46 (9.7)	146 (30.9)	473
Cervical	0	1072 (98.3)	0 (0.0)	17 (1.5)	0 (0.0)	2 (0.2)	1091
	I	187 (45.3)	2 (0.5)	196 (47.4)	26 (6.3)	2 (0.5)	413
	II	12 (2.8)	5 (1.2)	161 (38.2)	237 (56.2)	7 (1.6)	422
	III	15 (2.7)	11 (1.9)	168 (30.1)	352 (63.1)	12 (2.2)	558
	IV	5 (3.4)	8 (5.4)	67 (45.6)	61 (41.5)	6 (4.1)	147
Colorectal	0	18 (94.7)	0 (0.0)	1 (5.3)	0 (0.0)	0 (0.0)	19
	I	256 (61.4)	13 (3.1)	31 (7.4)	56 (13.4)	61 (14.7)	417
	II	329 (38.2)	208 (24.1)	73 (8.5)	145 (16.8)	107 (12.4)	862
	III	160 (24.5)	284 (43.6)	25 (3.8)	92 (14.1)	91 (14.0)	652
	IV	93 (15.5)	309 (51.6)	43 (7.2)	118 (19.7)	36 (6.0)	599
Lung	I	66 (68.0)	6 (6.2)	18 (18.6)	7 (7.2)	0 (0.0)	97
	II	20 (38.5)	14 (26.9)	9 (17.3)	9 (17.3)	0 (0.0)	52
	III	36 (3.8)	387 (40.8)	81 (8.5)	423 (44.5)	23 (2.4)	950
	IV	20 (2.1)	354 (36.6)	244 (25.2)	320 (33.1)	30 (3.1)	968
Stomach	0	8 (88.9)	0 (0.0)	1 (11.1)	0 (0.0)	0 (0.0)	9
	I	178 (93.7)	7 (3.7)	1 (0.5)	0 (0.0)	4 (2.1)	190
	II	93 (51.4)	38 (21.0)	23 (12.7)	3 (1.7)	24 (13.2)	181
	III	114 (33.0)	86 (24.9)	32 (9.3)	15 (4.3)	98 (28.5)	345
	IV	172 (23.2)	370 (49.9)	95 (12.8)	54 (7.3)	51 (6.8)	742
Oesophagus	0	4 (66.7)	0 (0.0)	2 (33.3)	0 (0.0)	0 (0.0)	6
	I	14 (70.0)	0 (0.0)	5 (25.0)	1 (5.0)	0 (0.0)	20
	II	32 (16.4)	8 (4.1)	97 (49.8)	56 (28.7)	2 (1.0)	195
	III	27 (7.8)	22 (6.3)	147 (42.2)	144 (41.4)	8 (2.3)	348
	IV	18 (6.3)	54 (19.0)	114 (40.2)	92 (32.4)	6 (2.1)	284
Oral cavity	0	11 (84.6)	0 (0.0)	2 (15.4)	0 (0.0)	0 (0.0)	13
	I	82 (72.6)	0 (0.0)	30 (26.5)	1 (0.9)	0 (0.0)	113
	II	73 (45.6)	2 (1.3)	69 (43.1)	14 (8.7)	2 (1.3)	160
	III	53 (28.1)	8 (4.2)	83 (43.9)	37 (19.6)	8 (4.2)	189
	IV	90 (23.9)	25 (6.6)	137 (36.5)	83 (22.1)	41 (10.9)	376

Considering tumours at all sites (but excluding nonmelanoma skin tumours), the survival rate was 57.1% for cases diagnosed between 2000 and 2005 (Table 3). The five-year survival rates were also calculated for each of the eight primary sites (Table 3). These calculations showed that among the most prevalent malignancies, the usually screened cancers had the highest survival rates: Prostate, breast and cervical cancer had survival rates of 78.2%, 74.8% and 60.2%, respectively. In contrast, lung and oesophagus cancer had five-year survival rates of 9.2% and 8.7%, respectively.

**Table 3:** Five-year specific survival for cancer cases diagnosed from 2000–2005

**Primary site**	**Total cases**	**Total events**	**Five-year specific survival (%)**
Prostate	2487	619	78.2
Breast	2158	637	74.8
Cervical	751	300	60.2
Colorectal	1009	526	48.1
Lung	959	790	9.2
Stomach	673	464	28.2
Oesophagus	444	365	8.7
Oral cavity	385	200	45.9
**All cases**	**8871**	**3901**	**57.1**

**Figure 2:**
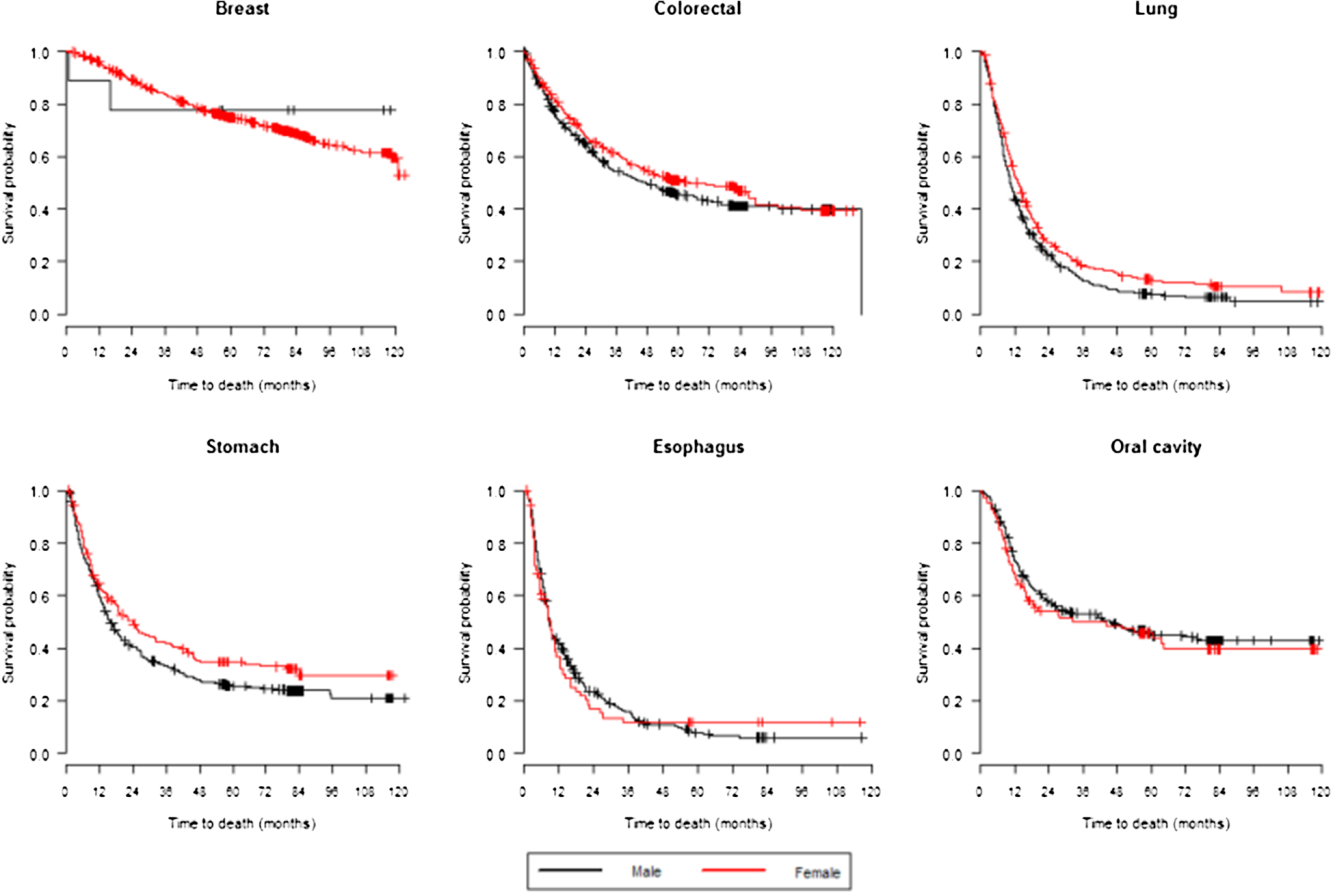
Survival curves according to sex as estimated using Kaplan-Meier methods.

**Figure 3:**
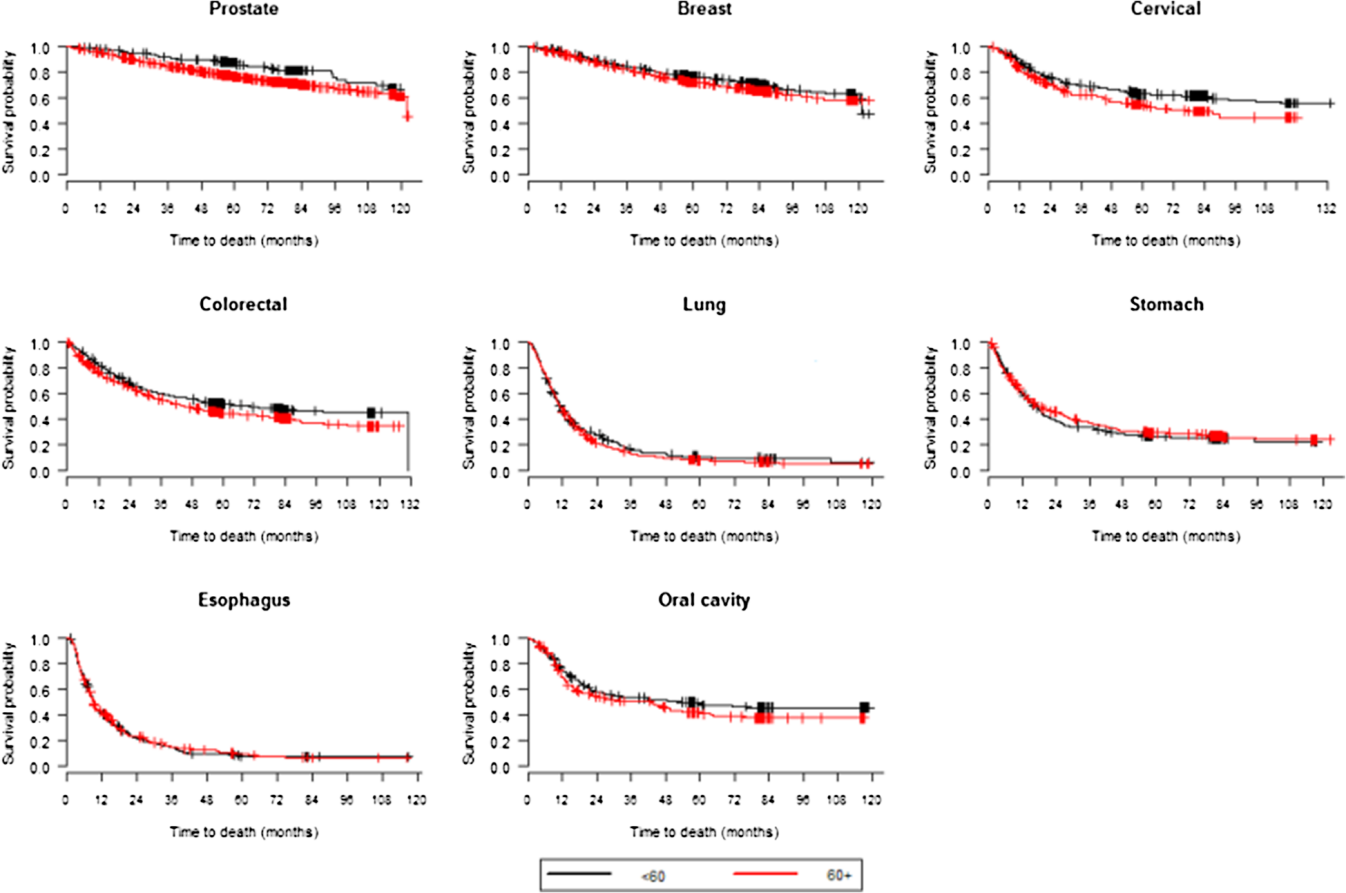
Survival curves according to age group as estimated using Kaplan-Meier methods.

**Figure 4:**
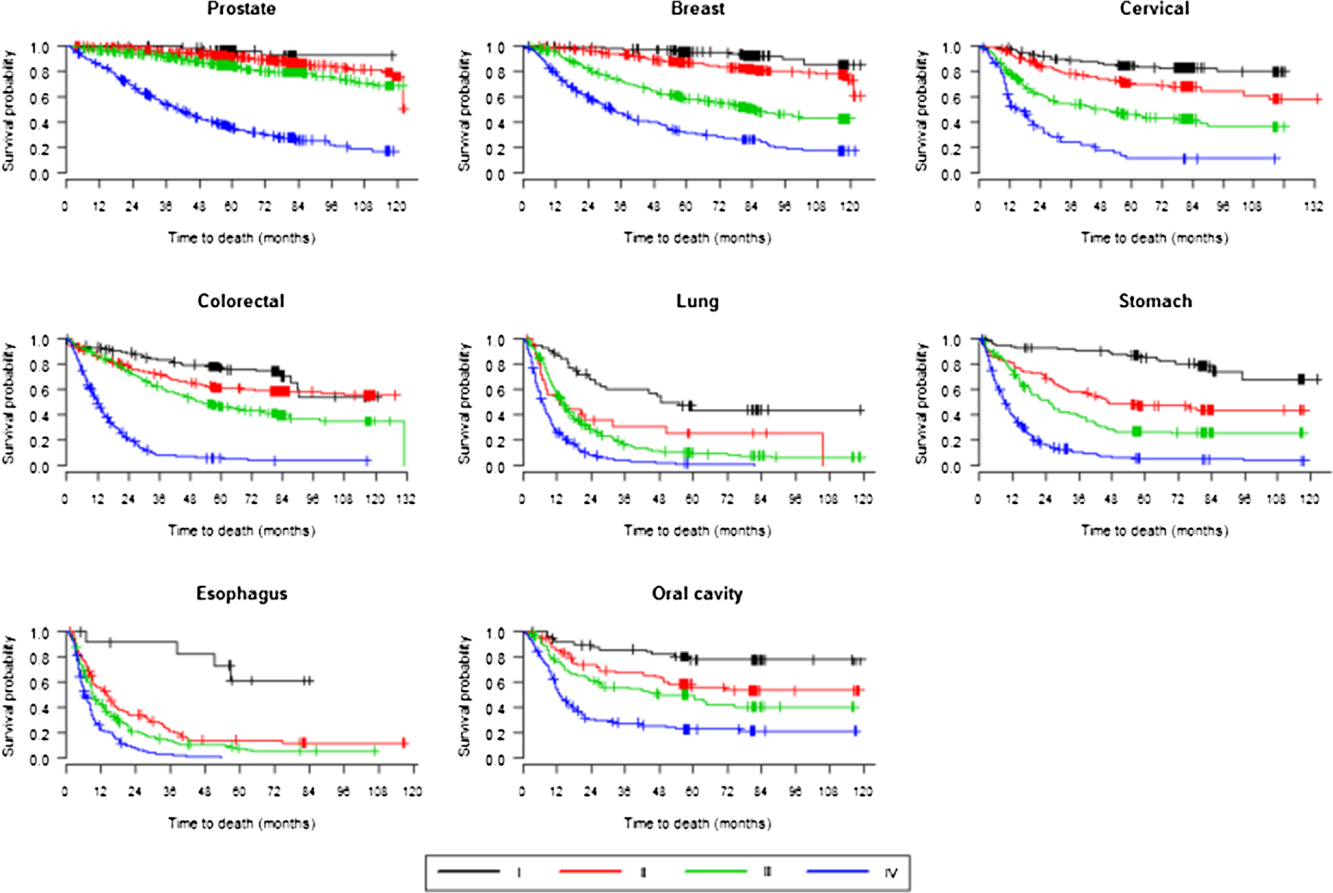
Survival curves according to clinical stage as estimated using Kaplan-Meier methods.

There were no significant differences in survival rates between men and women for the majority of cancers, even for breast tumours, which are rare for men (Table 4, Figure 2). Lung and stomach cancers had lower survival rates in men than in women (p<0.01 and p=0.03, respectively). For all tumours, except for stomach cancer and oesophagus cancer, patients who were younger than 60 years had higher survival rates than those who were 60+ years old (Table 4, Figure 3). Cases with clinical stage I tumours, including lung tumours, showed the highest survival rates regardless of other parameters (Table 4, Figure 4).

**Table 4:** Five-year specific survival (%) and log-rank test, according to sex, age group and clinical stage

**Primary site**	**Sex**		P-value*	**Age group**		P-value*	**Clinical stage**				P-value*
	**Male**	**Female**		**<60**	**60+**		**I**	**II**	**III**	**IV**	
Prostate	-	-	-	87.7	76.8	<0.01	96.5	91.6	84.4	36.0	<0.01
Breast	77.8	74.8	0.62	76.5	71.6	0.02	95.2	87.1	58.4	32.1	<0.01
Cervical	-	-	-	63.2	54.4	<0.01	79.7	61.5	38.5	11.2	<0.01
Colorectal	45.4	51.2	0.08	52.2	44.5	0.01	77.1	61.4	46.6	6.3	<0.01
Lung	7.6	12.9	<0.01	10.6	8.3	0.31	44.0	25.4	9.9	1.6	<0.01
Stomach	25.6	34.6	0.03	26.5	29.3	0.54	85.4	47.1	26.8	5.8	<0.01
Oesophagus	7.8	11.8	0.76	7.7	9.6	0.87	62.9	14.2	7.8	0.0	<0.01
Oral cavity	45.5	45.8	0.51	48.4	42.2	0.20	77.9	56.2	50.0	23.1	<0.01

* Log-rank test.

The results of the multivariable Cox regression analyses are shown in Table 5. The regression models provided results that were similar to those from the univariate test (Table 5). For prostate, breast, colorectal and oral cavity, being 60 years old or older was a risk factor for death. Being female appeared to be a protection factor for lung cancer. Clinical stage was a risk factor for death from cancer for all primary tumour sites studied.

**Table 5:** Cox proportional hazards model for cancer-specific mortality

**Variable**	**Primary site**							
	**Prostate**	**Breast**	**Cervical**	**Colorectal**	**Lung**	**Stomach**	**Oesophagus**	**Oral cavity**
	**HR (95% CI)**	**HR (95% CI)**	**HR (95% CI)**	**HR (95% CI)**	**HR (95% CI)**	**HR (95% CI)**	**HR (95% CI)**	**HR (95% CI)**
*Sex*								
Male	-	1.00	-	1.00	1.00	1.00	1.00	1.00
Female	-	2.46 (0.61; 9.93)	-	0.88 (0.74; 1.05)	0.78 (0.66; 0.91)	0.86 (0.70; 1.06)	1.03 (0.77; 1.38)	1.10 (0.79; 1.55)
*Age-group*								
<60	1.00	1.00	1.00	1.00	1.00	1.00	1.00	1.00
60+	1.49 (1.13; 1.98)	1.29 (1.10; 1.52)	1.04 (0.81; 1.32)	1.27 (1.07; 1.52)	1.10 (0.95; 1.28)	0.92 (0.75; 1.11)	1.11 (0.900; 1.38)	1.41 (1.06; 1.88)
*Stage disease*								
I	1.00	1.00	1.00	1.00	1.00	1.00	1.00	1.00
II	2.22 (0.71; 6.96)	2.48(1.59; 3.86)	1.99 (1.26; 3.15)	1.94 (1.36; 2.78)	1.59 (0.82; 3.07)	3.60 (2.09; 6.22)	4.98 (1.80; 13.82)	2.10 (1.07; 4.15)
III	3.79 (1.20; 11.90)	8.10 (5.22; 12.59)	4.91 (3.18; 7.57)	3.23 (2.26; 4.63)	2.24 (1.36; 3.68)	6.73 (3.99; 11.35)	7.26 (2.63; 20.07)	3.03 (1.57; 5.86)
IV	20.13 (6.44; 62.94)	18.30 (11.67; 28.71)	10.84 (6.73; 17.47)	13.44 (9.39; 19.24)	4.88 (2.95; 8.06)	15.93 (9.62; 26.37)	12.00 (4.29; 33.56)	6.34 (3.34; 12.02)

HR, hazard ratio; CI, confidence interval.

## Discussion

Cancer registries that are administrated by cancer hospitals have been criticized for the plethora of reporting differences that could bias the data reported by these institutions [5]. Prior to the year 2000, we also found it difficult to evaluate the mortality rates at the Hospital de Câncer de Barretos due to differences in the methods used to report cancer and patient data. We solved this problem by creating a modern cancer registry based on standardized data reporting. In addition, our team of professionals was trained to report complex information in a standardized, easy-to-understand manner. As a result, we were able to extract and analyse key information about cancer survival at our institute.

The Hospital de Câncer de Barretos treats cancer patients that come from more than 2000 cities in Brazil, including many from remote regions. The socioeconomic characteristics of patients treated in Barretos are especially variable as a result of the wide range of risk factors and cultural norms in different Brazilian populations. This makes an analysis such as this one particularly interesting but also introduces potential bias. The data extraction and analysis were done carefully with attention to detail to avoid discrepancies and misinterpretations. We chose to analyse survival by using data obtained between 2000 to 2009 to ensure that the information had been collected and stored according to a standardized protocol. Our results are robust, describing the most frequent malignancies we treat at our institution and highlighting data that may help us understand the courses of aggressive malignancies.

The high rates of cancer cases treated at the institution in advanced stage do not necessarily reflect the Brazilian population situation, because the hospital is a tertiary center in the country, where there are many regions with lack of resources, impeding them to promote the early detection, appropriate diagnostic and treatment to its population, making necessary to send this cases to an institution far from the original area. An additional factor is the high number of illiterate Brazilians (9.6% according to the Census of the Brazilian Institute of Geography and Statistics – IBGE - in 2010) [6] that may lead to low levels of basic hygiene and health care, reflecting in late diagnosis. The high rate of illiterate patients identified at the institution (22%) can be explained by the fact that the majority of patients seen at the institution live in small cities, where in Brazil are higher than the large cities [6]. In addition, many patients reside in small towns from Northeast and North of the country (15%), regions that still have significant rates of illiteracy, above the national average.

Cancer aggressiveness and, consequently, mortality rates are thought to be directly related to Public Health efforts to detect cancer at early stages [10]. Although the study does not allow this type of inference, it was noticed that tumors targets Government prevention programs, such as the cervix and breast cancer, were the ones that had more diagnoses at the initial phase of the disease, giving them a higher probability of a good outcome. This is reflected in the high number of surgeries performed as first-line therapy for these types of cancer. Therapeutic success is in part associated with early cancer detection.

Prevention programmes carried out by the Brazilian Government are mostly opportunistic and Hospital de Câncer de Barretos is one of the proponents institutions of prevention programs for skin cancer [11], cervix, prostate [12,13] and breast cancer [14] recognized in the country. The effectiveness of these programs is perceived through the large number of patients recruited from around the country that are sent to the institution for clinical and complementary examination and specialized treatment. However, this number is low compared to the number of cases that come for treatment out of prevention programs. It is expected that in long-term these programs will help increase the number of cases of cervix, breast and prostate cancer treated at the institution that have been diagnosed in the early stages of the disease.

Although the data from a hospital registry does not necessarily represent the incidence or prevalence of cancer in a population, in countries where information from population-based cancer registries are underutilized such as Brazil and others, these data can help identify emerging trends, especially in regions where a few large hospitals treat a high volume of referred patients.

## Conclusion

During the ten-year period from 2000–2009, the Hospital de Câncer de Barretos Registry collected, processed and analysed data from all cases treated at the institution. These data provide relevant information about patient and disease characteristics and patient survival.

## Competing interests

The authors declare that they have no competing interests.

## Authors’ contributions

ECC participated in the design of the study, in data collection, analysis and interpretation and helped draft the manuscript. ECM was involved in revising the manuscript. MAAA and RMD participated in data collection and critically reviewed the manuscript. ALF helped draft the manuscript and was involved in revising the manuscript. VLV participated in the design of the study and in data interpretation, helped draft the manuscript and was involved in revising the manuscript. All authors read and approved the final manuscript.
